# Report of the first clinical case of intestinal trichomoniasis caused by *Tritrichomonas foetus* in a cat with chronic diarrhoea in Brazil

**DOI:** 10.1186/s12917-017-1026-3

**Published:** 2017-04-17

**Authors:** Aline S. Hora, Samantha I. Miyashiro, Fabiana C. Cassiano, Paulo E. Brandão, Archivaldo Reche-Junior, Hilda F. J. Pena

**Affiliations:** 1 Department of Preventive Veterinary Medicine and Animal Health, College of Veterinary Medicine, University of São Paulo, São Paulo, SP Brazil; 2 Department of Veterinary Medicine, College of Veterinary Medicine, University of São Paulo, São Paulo, SP Brazil; 3 Veterinary Hospital, College of Veterinary Medicine, University of São Paulo, São Paulo, SP Brazil

**Keywords:** Feline Tritrichomonosis, Large bowel diarrhea, Enteric pathogen

## Abstract

**Background:**

*Tritrichomonas foetus* is an emergent and important enteric pathogen of cats, which causes prolonged diarrhoea in cats.

**Case presentation:**

This study describes a *T. foetus* infection in a seven-month-old, entire male domestic shorthair kitten with a six-month history of persistent large intestinal diarrhoea, faecal incontinence, prostration, apathy and weight loss. Parasites were microscopically observed and confirmed by PCR and DNA sequencing. Molecular analyses were carried out comparing the sequence obtained in this study with *T. foetus* and *T. suis*. Retrieved from GenBank. After treatment with ronidazole, the cat showed resolution of clinical signs.

**Conclusions:**

This is the first clinical case of *T. foetus* infection in a chronic diarrheic cat in Brazil and South America, confirming the presence of this pathogen in this part of the world and highlighting the importance of this protozoa being considered in the differential diagnosis of cats presenting diarrhoea of the large intestine. Our case report enriches our knowledge on the geographical distribution of *T. foetus* in cats in Brazil and provides further understanding of the clinical significance of feline intestinal trichomoniasis in this country.

**Electronic supplementary material:**

The online version of this article (doi:10.1186/s12917-017-1026-3) contains supplementary material, which is available to authorized users.

## Background

*Tritrichomonas foetus* is a protozoal parasite in the order *Trichomonadida*, and family *Trichomonadidae* (Phylum Parabasalia) which also includes the genera *Trichomonas*, *Ditrichomonas* and *Pentatrichomonas* [1]. Originally described as a parasite restricted to the urogenital tract of cattle, *T. foetus* has more recently been shown to infect the gastrointestinal tract of domestic cats [2]. *T. foetus* has been reported to be a naturally occurring pathogen of the large intestine of domestic cats that colonizes the ileum, cecum, and colon, and resides in close contact with the epithelium, causing chronic large bowel diarrhoea [3, 4].

*T. foetus* has been diagnosed in the domestic cat in many geographic regions including Europe (Austria, Finland, France, Germany, Greece, Italy, the Netherlands, Norway, Poland, Spain, Sweden, Switzerland, and the UK), North America (Canada and the USA), Australia/Oceania (Australia and New Zealand), and Asia (Japan and South Korea) [5]. In Brazil, to date, infection with *T. foetus* has been reported only in asymptomatic cats [6]. This case report describes a case of naturally occurring *T. foetus* infection in a kitten inhabiting in Brazil, São Paulo state and the molecular analysis of this strain.

## Case presentation

A seven-month-old, entire male domestic shorthair kitten was presented to the Veterinary Hospital of the School of Veterinary Medicine – University of São Paulo, Brazil, in October 2013. This cat had a six-month history of persistent large intestinal diarrhoea, faecal incontinence, prostration, apathy and weight loss. Previously to the cat presentation at the Veterinary Hospital of the School of Veterinary Medicine, the cat was presented to veterinary practitioners and several different anthelminthics, metronidazole and sulfadimetoxin (dosage not available) were blindly administrated without success. The cat had been confined indoor, and had contact with three asymptomatic cats and a dog. Unfortunately, it was not possible to evaluate the contacting animals due to low compliance of the owner.

At physical examination, the cat showed sensibility to abdominal palpation in the mesogastric region, thickened bowel loops with faecal content, inflammation at the anal region and a body temperature of 39.2 °C. Screening tests for feline leukaemia virus - FeLV and feline immunodeficiency virus - FIV (SNAP FIV/FeLV Combo Test, Idexx Laboratories) were performed and the results were negative.

Faecal flotation was negative for the presence of nematode eggs, *Giardia* species cysts and coccidian oocysts using sugar centrifugation flotation as previously described [7].

**Fig. 1:**
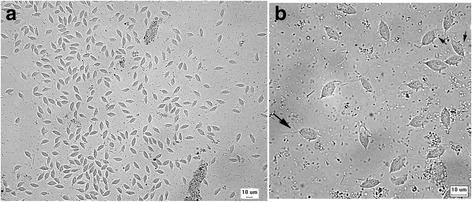
*Tritrichomonas foetus* in cat faeces. **a** Numerous pyriform trophozoites. **b** The three free anterior flagella (large arrow) and the undulating membrane (*small arrows*) can be visualised in some trophozoites. Fresh preparation in saline 0.85%



The fresh faecal sample obtained via colon flush was suspended in an equal volume of normal saline and microscopically examined under a cover slip using 200X and 400X magnification. *Tritrichomonas* sp. was identified by direct examination, which revealed very large numbers of motile trophozoites featuring a pyriform body, which measured approximately 10 to 20 μm in length, with three free anterior flagella, an undulating membrane running the length of the body and one posterior flagellum (Fig. 1a and b). The trophozoites exhibited jerky, progressive forward motion (Additional file 1: Movie S1). The motile characteristics of *T. foetus* differ from *Giardia* sp. in that a falling leaf motility has been observed [5].

**Additional file 1:** *Tritrichomonas foetus* in cat faeces. Fresh preparation in saline 0.85%. The trophozoites exhibited jerky, progressive forward motion. (MOV 39828 kb)


Both *T. foetus* and *Pentatrichomonas hominis* can occur in the intestinal tract of cats [6]. *P. hominis* lives as a mere commensal in the colon of cats [8] and a number of other mammalian hosts including humans, pigs, dogs, water buffalos, cows, and goats [9]. *Tritrichomonas* sp. and *Pentatrichomonas* sp. are not easily differentiated from one another by examining living material or organisms stained with routinely used histological stains [10]. Thus, molecular methods to detect *T. foetus* and *Pentatrichomonas* sp. were performed.

DNA was extracted directly from the fresh faecal sample using a commercial DNA extraction kit (DNeasy® Blood & Tissue, Qiagen), according to the manufacturer’s protocol. *T. foetus* and *P. hominis* were tested using the primers and conditions previously described by Felleisen et al. [11] and Crucitti et al. [12], respectively.

Coinfection with *T. foetus* and *P. hominis* was not observed herein, unlike the prior study conducted in Brazil, in which three of the four *T. foetus*-positive cats were infected with *P. hominis,* although both studies using the same set of primers for the molecular diagnosis of both infections [6]. The amplicon corresponding to *T. foetus* (347 bp) was purified using ExoSAP-IT PCR Product Cleanup (USB Products Affymetrix, Cleveland, OH) and submitted to bidirectional DNA sequencing with BigDye 3.1 (Applied Biosystems, Carlsbad, CA), according to the manufacturer’s protocols. The products were resolved using a 3500 Genetic Analyser (Applied Biosystems, Foster, CA), and the electropherograms were analysed with Phred at http://asparagin.cenargen.embrapa.br/phph/. Positions with a quality score of >20 were used to generate contiguous sequences with Cap-Contig implemented in the software Bioedit 7.0.9.0 [13]. The sequence was then submitted to BLAST/n at http://www.ncbi.nlm.nih.gov/BLAST to confirm the amplicon identities. Each sequence was aligned with homologous sequences from *T. foetus* retrieved from GenBank with CLUSTAL/W in Bioedit 7.0.9.0, and a phylogenetic tree for the nucleotide sequences was generated using the neighbor-joining distance algorithm and the maximum composite likelihood model with 1000 bootstrap replicates using MEGA 5.0 [14]. A sequence identity matrix was performed with Bioedit 7.0.9.0. The nucleotide sequence determined in this work was submitted to GenBank under accession number KU680816.

**Fig. 2:**
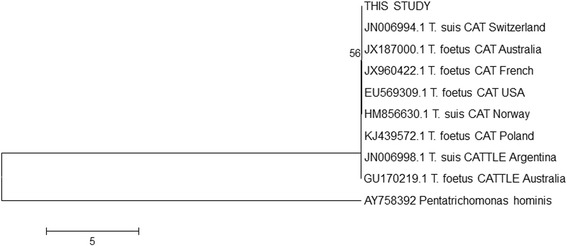
Neighbor-joining maximum composite likelihood phylogenetic tree of partial sequences of *Tritrichomonas foetus* and *T. suis* internal transcribed spacer 1 (partial), 5.8S ribosomal RNA gene (complete), and internal transcribed spacer 2 (partial). *Pentatrichomonas hominis* was used as an outgroup. The numbers on the nodes indicate the bootstrap support from 1000 replications. Only bootstrap values of >50 are shown. The scale bar represents the number of substitutions per nucleotide

Feline isolates and bovine isolates are morphologically indistinguishable [15]. Current evidence suggests that *T. foetus* and *T. suis* appear to correspond to the same species [15–18], as observed herein (Fig. 2). This finding is also sustained by a minor difference (0.04%) in identity in the sequences from *T. foetus* (KJ439572, GU170219) and *T. suis* (JN006998) retrieved from GenBank compared to the sequence from this study.

The kitten was treated with 30 mg/kg of ronidazole, administered orally once a day for 14 days, which resulted in improvement in faecal consistency and frequency without adverse effects. However, adverse effects, such as reversible neurological signs, including lethargy, trembling of extremities, ataxia, agitation, facial tremors and hyperesthesia, have already been observed in cats treated with this drug [19].

## Discussion and Conclusions

In recent years, *T. foetus* has become increasingly recognized as an important enteric pathogen of cats. A wide geographical distribution of this pathogen has been observed [5], in South America, *T. foetus* has previously been detected in asymptomatic cats in Brazil [6]. This is the first clinical case of intestinal trichomoniasis caused by *T. foetus* in Brazil and South America, confirming the presence of this pathogen in this part of the world and highlighting the importance of this protozoa being considered in the differential diagnosis of cats presenting diarrhoea of the large intestine. Our case report enriches our knowledge on the geographical distribution of *T. foetus* in cats in Brazil and provides further understanding of the clinical significance of feline intestinal trichomoniasis in this country.

## Abbreviations

FeLV: Feline leukaemia virus
FIV: Feline immunodeficiency virus
T. foetus: *Tritrichomonas foetus*
T. suis: *Tritrichomonas suis*
